# Associations between COVID-19 impact and subsequent substance use in adolescents with chronic pain

**DOI:** 10.3389/fpain.2025.1695346

**Published:** 2025-11-28

**Authors:** Bridget A. Nestor, Camila Koike, Kimberly Pokstis, Nicole Tacugue, Jack Dandaraw, Kristina Wright, Christine Greco, Elissa R. Weitzman, Lydia A. Shrier, Joe Kossowsky

**Affiliations:** 1 Department of Anesthesiology, Critical Care, and Pain Medicine, Boston Children’s Hospital, Boston, MA, United States; 2 Department of Psychology, Endicott College, Beverly, MA, United States; 3 Department of Anesthesia, Harvard Medical School, Boston, MA, United States; 4 Division of Adolescent/Young Adult Medicine, Boston Children’s Hospital, Boston, MA, United States; 5 Department of Pediatrics, Harvard Medical School, Boston, MA, United States; 6 Division of Sleep Medicine, Harvard Medical School, Boston, MA, United States

**Keywords:** COVID-19, adolescents, chronic pain, substance use, pain

## Abstract

**Objective:**

The current cross-sectional study retrospectively investigated associations between COVID-19-related factors and subsequent substance use in adolescents with chronic pain.

**Methods:**

A total of 243 adolescents with diagnosed pain disorders were retrospectively surveyed from September 2021 to May 2024. Descriptive statistics summarized past-month and past-year substance use; COVID-19 exposures, impact, and distress; mental health; and pain-related indicators. Logistic regressions estimated the odds of substance use based on COVID-19 exposures, impact, and distress, controlling for demographics, mental health, and pain.

**Results:**

Of the 243 adolescents (*M*_age_ = 16.9, SD = 1.42 years; 68.44% female), 39.9% reported past-year substance use, and 28.4% reported past-month substance use. All participants reported COVID-19 exposures (*M* = 9.68, SD = 3.53), impact (*M* = 34.00, SD = 10.11), and distress (*M* = 5.25, SD = 2.19). No differences in Exposures or Distress emerged between youth with vs. without substance use (*p*’s > 0.05). Youth with past-month (*U* = 2,522, *p* < 0.001) and past-year (*U* = 3,998, *p* < 0.001) substance use reported more Impact, compared with those without use. COVID-19 social impact predicted odds of past-year (OR = 1.25, 95% CI = 1.13–1.38) and past-month (OR = 1.27, 95% CI = 1.14–1.42) substance use, controlling for gender, anxiety, depression, stress, pain intensity, pain interference, and functional disability.

**Discussion:**

The social impact of COVID-19 uniquely predicted subsequent substance use, over and above mental and physical health symptoms in adolescents with chronic pain. Incorporating socially focused interventions into multidisciplinary pain treatment and prevention efforts may better support the health and wellness of youth with chronic pain.

## 1 Introduction

The COVID-19 pandemic affected adolescents worldwide through school shutdowns, activity cancellations, and social distancing orders, and it was associated with significant increases in youth anxiety, depression, irritability, and anger (1). For youth with chronic pain, a condition that impacts nearly 40% of children and adolescents globally, the initial pandemic lockdown was associated with mixed effects. For example, although youth with chronic pain reported more school-related difficulties and reduced time with peers, they also reported decreases in pain, pain catastrophizing, and corresponding reductions in psychological stress and anxiety (2). These positive byproducts of lockdown have generally been attributed to reduced demands associated with in-person schooling, which are often particularly taxing for youth with pain disorders.

Adolescent substance use is another important correlate and consequence of the COVID-19 pandemic. In samples of youth without chronic pain, overall substance use generally decreased for most substances during the pandemic lockdown, except for cannabis and alcohol use, which increased (3). Substance use was more likely in the face of increased mental health challenges, including depression and anxiety, familial hardship, and parental substance use (4), prior to the pandemic (5, 6). In the throes of social distancing orders, youth continued to report solitary, virtual, and even in-person substance use with peers (3).

For youth with chronic pain, substance use poses specific challenges as pain and substance use can bidirectionally reinforce and exacerbate each other (7), and youth with pain often use substances to help manage symptoms of their pain (8, 9). Yet, how the pandemic affected longer-term trends of substance use in youth with chronic pain has been considerably less studied. Cross-sectional work in youth with pain during the pandemic has shown that more than half endorsed increases in alcohol, tobacco, vaping, and other substance use (2), and concerningly, upticks in substance use emergency department visits were also observed (10). Other work in young adults with chronic pain identified stable rates of substance use from pre- to during the pandemic (11). These studies, however, did not investigate the longitudinal effects of COVID-19 on substance use outcomes in adolescents with pain.

Identifying COVID-19 predictors of substance use in youth with chronic pain is critical not only for understanding the lasting impacts of the pandemic but also because this population is vulnerable to engaging in ongoing, potentially ineffective, instrumental substance use (8, 9). In addition, identifying potential risk factors for substance use that were present during the pandemic but may also emerge in non-pandemic contexts (e.g., emotional distress, withdrawal from social activity, loss of routine, etc.) can help guide treatment and prevention efforts in these youth. Understanding these long-term effects may help tailor support measures for youth with chronic pain in the event of similar, albeit less intense, circumstances (e.g., short-term school absences or health setbacks).

The current study therefore aimed to investigate the effects of COVID-19 exposure, impact, and distress on substance use in adolescents with chronic pain. We hypothesized that increased exposures to COVID-19-related events, increased COVID-19-related impact on well-being, and increased COVID-19-related distress would predict increased odds of substance use across substances. Specifically, in line with prior work linking emotional distress to adolescent substance use (6, 12–14), we expected that the emotional impact of the pandemic would most predict substance use. We further explored whether these associations remained significant after accounting for demographic, psychological, and pain-related variables.

## 2 Materials and methods

### 2.1 Participants and procedures

Participants were recruited from a pediatric pain clinic in the Northeast USA between September 2021 and May 2024. Eligible adolescents were between 14 and 19 years old, English-speaking, and actively receiving care for a diagnosed chronic pain condition. Patients with severe cognitive impairment were excluded. Potential participants were identified through weekly chart reviews. Eligible patients and their families were contacted via mail, email, or secure patient portal messaging. Consent (from parents/guardians) and assent (from adolescents) were obtained online or in the clinic. Participants completed the survey electronically using REDCap, a secure HIPAA-compliant platform. This study was cross-sectional, and participants provided both current and retrospective reports on their functioning in the survey. The study was approved by the Boston Children's Hospital IRB (IRB-P00036526), and participants received a $20 gift card for their time.

### 2.2 Measures

#### 2.2.1 Demographics

Questions assessing demographics collected information regarding participants’ age, grade, race, ethnicity, and self-reported gender.

#### 2.2.2 COVID-19 exposure and impact

The **COVID-19 Exposure and Family Impact Scales Adolescent and Young Adult Version (CEFIS-AYA)** (15) is a validated instrument designed to assess the impact of the COVID-19 pandemic on families of children with pediatric health conditions across three domains: **CEFIS Exposure** consists of 32 yes/no items measuring direct and indirect exposures to COVID-19 (e.g., illness, school disruption) from March 2020 (pandemic onset) to present. The total score is the sum of “yes” responses (range: 0–32). **CEFIS Impact** consists of 15 items rated on a 4-point Likert scale (1, made it a lot better; 4, made it a lot worse). Subscales include **Physical Well-Being** (six items; range: 6–24), **Emotional Well-Being** (four items; range: 4–16), and **Social Well-Being** (five items; range: 5–20). These were summed to create a total CEFIS Impact score (range: 15–60). **CEFIS Distress** uses a single-item 10-point scale assessing subjective COVID-19-related distress (higher scores = greater distress).

#### 2.2.3 Substance Use

**Substance use** was assessed using items adapted from validated surveys (14, 16–19). Participants reported past-year and past-month use of cannabis, alcohol, tobacco, vaping, and non-medical opioids. Those who endorsed lifetime use completed items quantifying how frequently (ranging from 0 to more than 40 occasions) they had used each substance in the past year and past month. Participants who reported more than 0 occasions of substance use within the past 12 months or past 30 days were classified for past-year and past-month substance use accordingly.

#### 2.2.4 Psychological variables

**Anxiety and depressive symptoms** were assessed using the Patient-Reported Outcomes Measurement Information System (PROMIS) Anxiety and Depression Short Forms (20–22). PROMIS consists of validated self-report instruments designed to evaluate various health-related domains. Specifically, the anxiety and depression measures utilized an eight-item questionnaire assessing the frequency of certain feelings or behaviors over the previous week. Responses were scored using a 5-point Likert scale (*1, never*; *5, almost always/always*). Raw scores were summed and then transformed into standardized *T*-scores, with higher scores indicating more severe anxiety and depressive symptoms. Participants completed either the pediatric (ages 14–17) or adult (ages 18–19) version based on their age; their scores were combined by converting them into *z*-scores. Internal consistency reliability for these measures was excellent, as indicated by Cronbach's alpha coefficients ranging from 0.93 for anxiety and 0.96 for the depressive symptoms scale.

**Psychological stress** was evaluated using the PROMIS Psychological Stress Short Form (23, 24). This measure consisted of a four-item questionnaire assessing the frequency of specific feelings or behaviors experienced during the past week. Items were scored on a 5-point Likert scale (*1, never*; *5, almost always/always*). Raw scores were summed and converted into standardized *T*-scores, with higher *T*-scores indicating higher psychological stress. Participants completed the pediatric version of this measure. Internal consistency reliability was excellent, with Cronbach's alpha at 0.92 across all assessments.

#### 2.2.5 Pain variables

**Pain intensity** was measured using the numerical rating scale (25) (NRS) on a 0–10 continuous scale (0, *no pain*; 10, *worst pain imaginable*).

**Functional disability** was assessed by the functional disability inventory (26) (FDI) is a validated self-report instrument comprising 15 items examining perceived difficulty related to physical activity. Participants rated perceived difficulty of activities at home, school, or recreational settings using a 5-point Likert scale (0, *no trouble*; 4, *impossible*). Raw scores were summed to calculate a total score (0–60), with higher scores indicating more severe pain-related functional disability. Cronbach's alpha in the current study was 0.91, suggesting excellent internal consistency reliability.

**Pain interference** was assessed by the Patient-Reported Outcomes Measurement Information System (PROMIS) Pain Interference (22, 27) comprises eight items measuring the frequency with which an action or feeling occurred in the prior week. A 5-point Likert scale was used to score responses (1, *never*; *5*, *almost always/always*). Raw scores were summed and converted into standardized *T*-scores. Higher *T*-scores are indicative of greater occurrences of the examined domain. Participants completed either the pediatric version (ages 14–17) or the adult version (ages 18–19), and their scores were combined by converting them into *z*-scores.

### 2.3 Data analysis

Statistical analyses were performed in Python version 3.9.6 (28) and SPSS version 28 (29). Participants were categorized into two substance use groups based on any reported substance use (yes/no) for the past year and past month. Due to violations of normality and homogeneity assumptions (via Shapiro–Wilk and Levene's tests), non-parametric tests were used: Mann–Whitney *U* tests for continuous variables and Monte Carlo-based chi-square tests for categorical comparisons. For measures with specified age cutoffs (i.e., PROMIS Pain Interference, Anxiety, and Depressive Symptoms), descriptive analyses and tests of group differences based on substance use were conducted separately for pediatric-aged (14–17) and adult-aged (18–19) participants. Spearman correlations were computed across all key variables. False discovery rate (FDR) correction was applied for multiple comparisons, and Winsorization was used to reduce the influence of extreme outliers.

To identify predictors of substance use, we conducted a series of five hierarchical logistic regression models (see Table 1). Variables were added in blocks: (1) total CEFIS scores, (2) CEFIS Impact subscales, (3) demographics, (4) psychological variables, and (5) pain-related factors. Predictors that reached statistical significance at one step were retained in subsequent models. Statistical significance was defined as *p* < 0.05, and 95% confidence intervals (CI) were reported for all odds ratios (ORs).

**Table 1:** Logistic regression models.

Variable	Model 0	Model 1	Model 2	Model 3	Model 4
Predictors	CEFIS total scores for exposure, impact, and distress	CEFIS Impact subscales (emotional, physical, social)	Demographic covariates (age, gender)	Psychological variables (psychological stress, anxiety, depressive symptoms)	Pain-related variables (pain intensity, functional disability, pain interference)

CEFIS scores: COVID-19 exposure and family impact scales adolescent and young adult version. Functional disability, functional disability scale; psychological stress, PROMIS psychological stress; pain intensity, numerical rating scale; pain interference, PROMIS pain interference pediatric and adult versions; anxiety, PROMIS anxiety pediatric and adult versions; depression, PROMIS depression pediatric and adult versions.

## 3 Results

Of the 312 patients approached, 40 did not complete the survey, and 29 were excluded due to incomplete data. The final analysis included 243 adolescents with complete data. Participants had a mean age of 16.9 (SD = 1.42) years, and 68.44% were female. Substance use was common: 39.9% (97/243) reported past-year substance use, 28.4% (69/243) past-month substance use, and 57.20% (139/243) no substance use (non-SU). See Table 2 and Supplementary Table S1 for additional demographic information.

**Table 2:** Demographic characteristics.

Variable	Total (*N* = 243)	Non-SU^a^^,^^b^ (*n* = 139)	Past-year SU^a^ (*n* = 97)	Past-month SU^b^ (*n* = 69)	*U*, *χ*^2^	*p* ^a^	*U*, *χ*^2^	*p* ^b^
Age, mean (SD)	16.9 (1.42)	16.53 (1.45)	17.4 (1.2)	17.53 (1.12)	4,429.00	<0.001*	2,803.00	<0.001*
Self-reported gender, *n* (%)								
Female	167 (68.72)	102 (73.38)	60 (61.86)	44 (63.77)	11.16	0.18	10.57	0.22
Male	36 (14.81)	17 (12.23)	17 (17.53)	11 (15.94)				
Non-binary	24 (9.88)	9 (6.48)	15 (15.46)	11 (15.94)				
Other	16 (6.59)	11 (7.91)	5 (5.15)	3 (4.35)				
Ethnicity, *n* (%)								
Hispanic or Latino, *n* (%)	19 (7.82)	10 (7.19)	7 (7.22)	4 (5.80)	7.32	0.11	5.86	0.19
Race, *n* (%)								
Asian	3 (1.23)	1 (0.72)	2 (2.06)	2 (2.90)	40.32	0.01*	10.03	0.26
Black or African American	3 (1.23)	2 (1.44)	1 (1.03)	1 (1.45)				
White	200 (82.31)	118 (84.89)	79 (81.45)	55 (79.71)				
Other Race/multiracial	37 (15.23)	18 (12.95)	15 (15.46)	11 (15.94)				
Reported substance used, *n* (%)								
Cannabis	NA	NA	56 (57.73)	40 (57.97)	NA	NA	NA	NA
Alcohol	NA	NA	74 (76.28)	40 (57.97)	NA	NA	NA	NA
Vape	NA	NA	34 (35.05)	19 (27.53)	NA	NA	NA	NA
Tobacco smoke	NA	NA	11 (11.34)	3 (4.35)	NA	NA	NA	NA
Opioids	NA	NA	13 (13.40)	5 (7.24)	NA	NA	NA	NA
CEFIS scores, mean (SD)								
Total exposure	9.68 (3.53)	9.43 (3.55)	9.99 (3.56)	9.97 (3.52)	6,114.50	0.26	4,410.50	0.39
Total impact	34.00 (10.11)	31.90 (9.83)	37.64 (8.54)	38.67 (8.20)	3,998.00	<0.001*	2,522.00	<0.001*
Total distress	5.25 (2.19)	5.07 (2.23)	5.49 (2.09)	5.47 (2.19)	5,882.00	0.12	4,183.50	0.18
Physical impact	14.23 (4.56)	13.45 (4.45)	15.45 (4.33)	15.89 (4.36)	4,669.50	0.001*	3,009.00	0.001*
Emotional impact	11.78 (3.24)	11.36 (3.41)	12.40 (2.69)	12.56 (2.55)	5,298.50	0.03*	3,593.50	0.03*
Social impact	8.54 (3.37)	7.66 (3.31)	9.89 (2.92)	10.21 (2.86)	3,631.50	<0.001*	2,365.50	<0.001*
Psychological variables, mean (SD)								
Depressive Symptoms (pediatric)	56.89 (11.64)	54.96 (12.69)	60.80 (8.14)	61.78 (7.43)	2,721.00	0.001*	1,803.00	0.001*
Depressive Symptoms (adult)	55.42 (9.17)	52.64 (9.95)	56.25 (7.81)	56.43 (8.33)	367.50	0.20	270.00	0.216
Anxiety (pediatric)	56.53 (11.52)	55.59 (12.18)	58.18 (10.19)	56.43 (8.33)	3,254.00	0.09	2,171.00	0.05*
Anxiety (adult)	57.38 (10.42)	53.35 (11.10)	60.03 (8.78)	56.43 (8.33)	299.50	0.04*	230.00	0.07
Psychological stress	62.20 (9.59)	60.78 (10.31)	64.23 (8.45)	64.41 (8.24)	5,428.00	0.01*	3,823.00	0.02*
Pain variables, mean (SD)								
Functional disability	23.97 (11.58)	23.46 (12.14)	24.02 (10.68)	23.78 (10.86)	6,242.00	0.70	4,477.00	0.84
Pain interference (pediatric)	60.26 (7.00)	59.89 (7.42)	60.62 (6.14)	60.70 (6.09)	3,626.50	0.54	2,542.50	0.53
Pain interference (adult)	59.97 (8.17)	56.52 (9.08)	61.52 (6.36)	61.61 (6.85)	324.50	0.06	240.00	0.07
Pain intensity	5.54 (1.68)	5.69 (1.74)	5.30 (1.54)	5.46 (1.54)	7,447.00	0.09	5,030.00	0.39

Numerical data differences were calculated using Mann–Whitney *U* test and categorical data using the Monte Carlo chi-square. Significant *p* < 0.05.

The *z*-score was calculated to combine the PROMIS pain interference, anxiety, and depression pediatric and adult versions.

*p*, *p*-value; SD, standard deviation; SU, substance use; *U*, *U*-statistic; *N*, total population; *n*, population; χ^2^, chi-square statistic.

CEFIS scores, COVID-19 exposure and family impact scales adolescent and young adult version. Functional disability, functional disability scale; psychological Stress, PROMIS psychological stress; pain intensity, numerical rating scale; pain interference, PROMIS pain interference pediatric and adult versions; anxiety, PROMIS anxiety pediatric and adult versions; depression, PROMIS depression pediatric and adult versions.

a Past-year SU vs. non-SU.

b Past-month SU vs. non-SU.

*Indicates significant *p*-values (*p*-value < 0.05).

Compared with adolescents with non-SU, those reporting past-year or past-month substance use were significantly older (*p*’s < 0.001) and had a lower proportion identifying as White (*p* = 0.01 for past-year use). Additionally, pediatric-aged participants who reported past-year or past-month substance use had significantly higher levels of depressive symptoms (*p*’s = 0.001) and psychological stress (*p* = 0.01 for past-year; *p* = 0.02 for past-month) compared with non-SU. Furthermore, higher levels of anxiety were observed in adult-aged participants with past-year (*p* = 0.04) use and pediatric-aged participants with past-month use (*p* = 0.05). Participants reporting substance use also indicated significantly greater overall COVID-19 impact (*p*’s < 0.001), including higher scores specifically in the physical (*p*’s = 0.001), emotional (*p*’s = 0.03), and social (*p*’s < 0.001) impact domains (Table 2).

**Figure 1:**
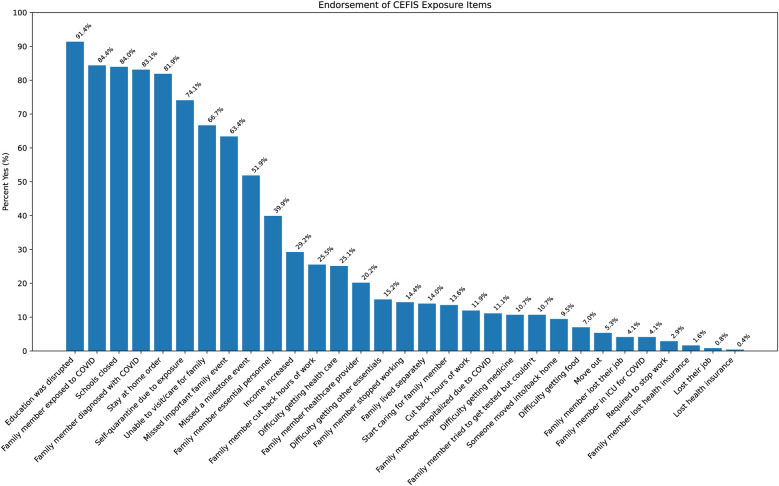
Endorsement of CEFIS Exposure items. CEFIS scores: COVID-19 exposure and family impact scales adolescent and young adult version.

**Figure 2:**
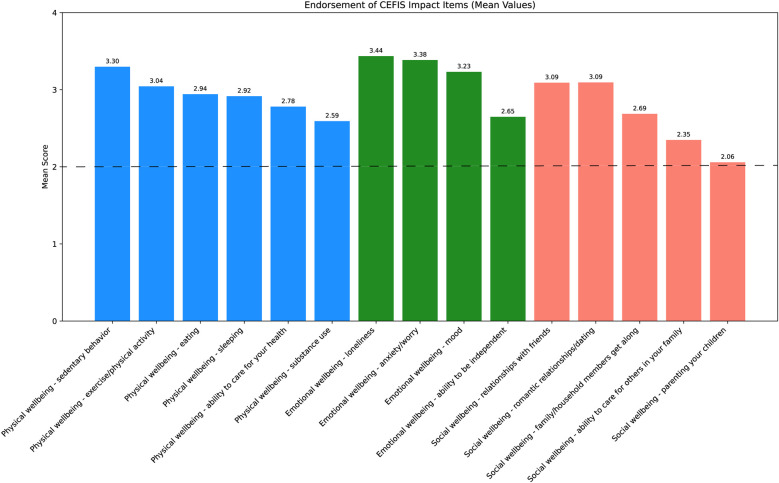
Endorsement of CEFIS Impact items. Range scores: (1) made it a lot better; (2) made it a little better; (3) made it a little worse; (4) made it a lot worse. The dotted line indicates the mean score of 2; scores above this line reflect a negative impact, while scores below indicate a positive impact. CEFIS, COVID-19 exposure and family impact scales adolescent and young adult version.

Analyses of the CEFIS Exposure items revealed only one significant group difference: adolescents in both the past-year and past-month SU groups were more likely to endorse “Had to cut back hours at work” compared with those with non-SU (*p*’*s* = 0.01) (Supplementary Table S2). No other exposure items differed between groups. Detailed responses of all CEFIS Exposure items are visualized in Figures 1 and 2. Additional information on the CEFIS Impact–Physical domain is presented in Supplementary Figure S1.

### 3.1 Examining correlations between CEFIS scores and substance use

**Figure 3:**
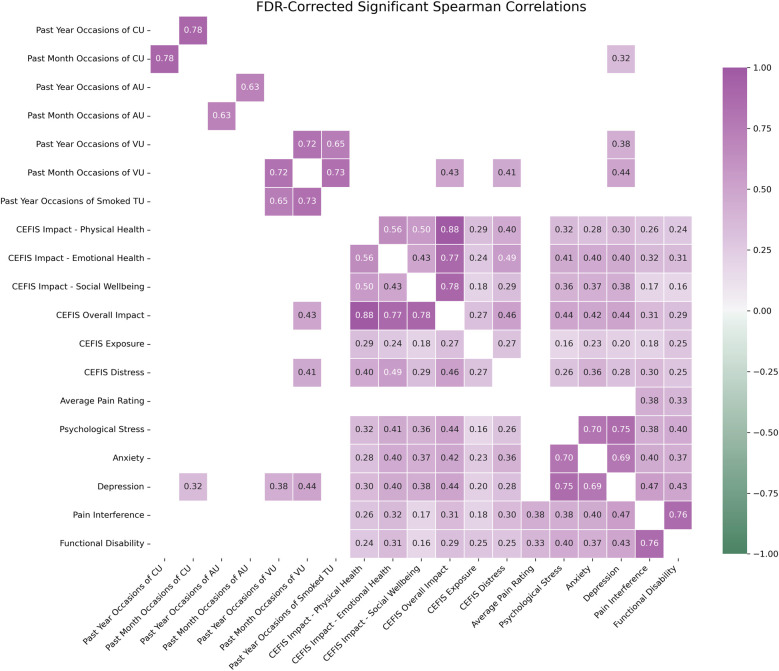
Spearman correlation matrix of entire sample (*n* = 243). Only FDR-corrected statistically significant correlation pairs (*p* < 0.05) are shown in the heatmap. CEFIS scores: COVID-19 exposure and family impact scales adolescent and young adult version. CU, cannabis use; AU, alcohol use; VU, vape use; TU, tobacco use; functional disability, functional disability scale; psychological stress, PROMIS psychological stress; pain intensity, numerical rating scale; pain interference, PROMIS pain interference pediatric and adult versions; anxiety, PROMIS anxiety pediatric and adult versions; depression, PROMIS depression pediatric and adult versions. *z*-scores were calculated to combine the PROMIS pain interference, anxiety, and depression pediatric and adult versions.

Correlation analyses across the total sample between CEFIS variables and substance use indicated significant positive associations between past-month occasions of vaping and CEFIS Distress (*r* = 0.41, *p* = 0.03) as well as CEFIS Impact (*r* = 0.43, *p* = 0.02). See Figure 3 and Supplementary Text S1 for all correlation results.

### 3.2 Logistic regression: past-year and past-month substance use

**Figure 4:**
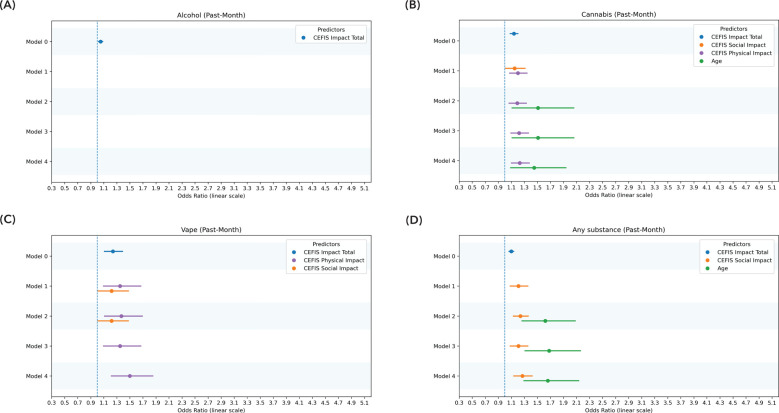
Significant predictors of past-month use: logistic regression model. **(A)** Significant predictors of past-month alcohol use. **(B)** Significant predictors of past-month cannabis use. **(C)** Significant predictors of past-month vaping. **(D)** Significant predictors of past-month any substance use. Model 0: CEFIS total scores for exposure, impact, and distress. Model 1: CEFIS Impact subscales (emotional, physical, social). Model 2: demographic covariates (age, gender). Model 3: psychological variables (psychological stress, anxiety, depressive symptoms). Model 4: pain-related variables (pain intensity, functional disability, pain interference). CEFIS scores: COVID-19 exposure and family impact scales adolescent and young adult version.

**Figure 5:**
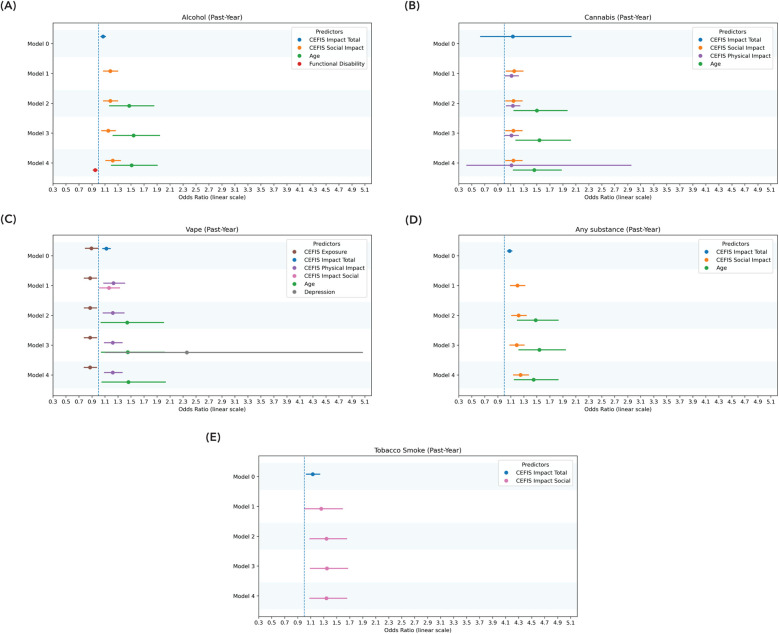
Significant predictors of past-year use: logistic regression model. **(A)** Significant predictors of past-year alcohol use. **(B)** Significant predictors of past-year cannabis use. **(C)** Significant predictors of past-year vaping. **(D)** Significant predictors of past-year any substance use. **(E)** Significant predictors of past-year tobacco smoke. Model 0: CEFIS total scores for exposure, impact, and distress. Model 1: CEFIS Impact subscales (emotional, physical, social). Model 2: demographic covariates (age, gender). Model 3: psychological variables (psychological stress, anxiety, depressive symptoms). Model 4: pain-related variables (pain intensity, functional disability, pain interference). CEFIS Scores: COVID-19 exposure and family impact scales adolescent and young adult version. Functional disability, functional disability scale; depression, PROMIS depression pediatric and adult versions. The *z*-score was calculated to combine the PROMIS depression pediatric and adult versions.

Full logistic regression model results, including odds ratios, standard errors, and significance values across all five modeling steps and substance use outcomes, are presented in Supplementary Tables S3–S12. Significant regression model results are presented in Figures 4 and 5. Below, we summarize the final models highlighting predictors that remained significant after controlling for demographic, psychological, and pain-related variables. In predicting past-year substance use, CEFIS Impact–Social remained a significant predictor (OR = 1.25, 95% CI: 1.13–1.38, *p* < 0.001) after adjusting for age, which was also significantly associated with substance use (*p* = 0.001). Functional disability (*p* = 0.37), pain intensity (*p* = 0.16), and pain interference (*p* = 0.34) did not significantly predict past-year use after accounting for CEFIS Impact–Social and age. The final model was significant, *χ*^2^(5) = 45.01, *p* < 0.001, Nagelkerke *R*^2^ = 0.24 (Figure 5 and Supplementary Table S3).

Similarly, for past-month substance use, the CEFIS Impact–Social was again significantly predictive (OR = 1.27, 95% CI: 1.14–1.42, *p* < 0.001), with age remaining a significant covariate (*p* < 0.001). Functional disability (*p* = 0.22), pain intensity (*p* = 0.64), and pain interference (*p* = 0.61) were not significant predictors. The final past-month model was significant, *χ*^2^(5) = 44.36, *p* < 0.001, Nagelkerke *R*^2^ = 0.26 (Figure 4 and Supplementary Table S4).

### 3.3 Logistic regression: alcohol use

The CEFIS Impact–Social (OR = 1.22, 95% CI: 1.11–1.35, *p* < 0.001) was significantly predictive of past-year alcohol use (OR = 1.51, 95% CI: 1.19–1.92, *p* < 0.001), with age remaining a significant covariate. Functional disability (OR = 0.95, 95% CI: 0.91–0.99, *p* = 0.02), pain intensity (*p* = 0.67), and pain interference (*p* = 0.39) were not significant predictors. The final past-year alcohol use model was significant, *χ*^2^(5) = 37.93, *p* < 0.001, Nagelkerke *R*^2^ = 0.22. None of the CEFIS components significantly predicted past-month alcohol use (Figures 4, 5 and Supplementary Tables S7, S8).

### 3.4 Logistic regression: cannabis use

The CEFIS Impact–Social (OR = 1.14, 95% CI: 1.02–1.28, *p* = 0.02) and CEFIS Impact–Physical (OR = 1.11, 95% CI: 1.01–1.22, *p* = 0.03) were significantly predictive of past-year cannabis use (OR = 1.46, 95% CI: 1.13–1.90, *p* = 0.004), with age also remaining a significant covariate. Functional disability (*p* = 0.57), pain intensity (*p* = 0.53), and pain interference (*p* = 0.39) were not significant predictors. The final past-year cannabis use model was significant, *χ*^2^(6) = 35.06, *p* < 0.001, Nagelkerke *R*^2^ = 0.22 (Figure 5 and Supplementary Table S5).

Past-month cannabis use was predicted by CEFIS Impact–Physical (OR = 1.23, 95% CI: 1.10–1.37, *p* < 0.001), with age remaining a significant covariate (OR = 0.45, 95% CI: 1.08–1.96, *p* = 0.01). Functional disability (*p* = 0.34), pain intensity (*p* = 0.76), and pain interference (*p* = 0.16) were not significant predictors. The final past-month model was significant, *χ*^2^(5) = 32.53, *p* < 0.001, Nagelkerke *R*^2^ = 0.22 (Figure 4 and Supplementary Table S6).

### 3.5 Logistic regression: vape

CEFIS Exposure total (OR = 0.87, 95% CI: 0.78–0.99, *p* = 0.03) and CEFIS Impact–Physical (OR = 1.22, 95% CI: 1.10–1.38, *p* = 0.002) were significantly predictive of past-year vaping, with age remaining a significant covariate (OR = 1.46, 95% CI: 1.05–2.03, *p* = 0.03). Functional disability (*p* = 0.43), pain intensity (*p* = 0.39), and pain interference (*p* = 0.92) were not significant predictors. The past-year vaping model was significant, *χ*^2^(7) = 28.37, *p* < 0.001, Nagelkerke *R*^2^ = 0.21 (Figure 5 and Supplementary Table S9).

CEFIS Impact–Physical was significantly predictive (OR = 1.50, 95% CI: 1.20–1.84, *p* < 0.001) of past-month vaping. Functional disability (*p* = 0.78), pain intensity (*p* = 0.08), and pain interference (*p* = 0.81) were not significant predictors. The final past-month vaping model was significant, *χ*^2^(4) = 26.79, *p* < 0.001, Nagelkerke *R*^2^ = 0.27 (Figure 4 and Supplementary Table S10).

### 3.6 Logistic regression: tobacco smoke

CEFIS Impact–Social was significantly predictive (OR = 0.34, 95% CI: 1.09–1.65, *p* = 0.006) of past-year tobacco smoke. Functional disability (*p* = 0.71), pain intensity (*p* = 0.89), and pain interference (*p* = 0.20) were not significant predictors. The past-year tobacco smoke model was significant, *χ*^2^(4) = 11.95, *p* = 0.02, Nagelkerke *R*^2^ = 0.16. None of the CEFIS components significantly predicted past-month tobacco smoke (Figure 5 and Supplementary Tables S11, S12).

## 4 Discussion

The current study investigated associations between COVID-19 and subsequent substance use in adolescents with chronic pain. Consistent with hypotheses, findings indicated greater reported COVID-19 impact was associated with increased odds of past-year and past-month substance use. In contrast with our hypotheses, however, the social impact of COVID-19, rather than the emotional impact, was a robust predictor of substance use, over and above the influence of both mental health and physical health concerns. These findings highlight the unique impact of COVID-19-related interpersonal stressors on subsequent substance use, suggesting critical risk factors for a population already vulnerable to substance use behavior.

While we broadly anticipated that the COVID-19 impact would be associated with increased substance use, we expected that the emotionally specific impact would be most tied to substance use outcomes, given prior evidence associating negative affect and mental distress with substance use in adolescents (3, 5, 10). However, our findings indicated a stronger role for social disruptions, particularly relationship deterioration among peers and friends. Adolescents in our sample rated disruptions to friendships as particularly distressing, suggesting that peer-related social disruptions might uniquely drive substance use. This finding aligns with broader adolescent literature emphasizing the predictive strength of peer relationship difficulties for substance use behaviors (30). This finding should also be considered in the context of normative developmental milestones of adolescence that were disrupted during the pandemic including cultivating peer relationships, forming identity, and establishing autonomy and separation from parents (31, 32). Notably, our results contrast somewhat with other studies suggesting that reduced social interactions were associated with lower adolescent substance use (33). It is therefore possible that interpersonal conflicts or perceived relational strain, rather than mere isolation or reduced social contact, more clearly drive substance use in adolescents with chronic pain. Additionally, older age consistently predicted increased likelihood of substance use, in line with developmental literature indicating heightened risk during later adolescence (19).

In terms of specific substance use, consistent with our main results, COVID-19 social impact significantly predicted past-year alcohol and tobacco use. Interestingly, physical impact, reflecting disruptions in exercise, sedentary behavior, sleep, and health self-management, significantly predicted past-year and past-month cannabis and vaping use. These findings suggest that adolescents with chronic pain may select substances based on perceived instrumental benefits, such as cannabis or vaping for managing physical health complaints, pain, or sleep disturbances, consistent with prior studies on instrumental substance use for symptom management (8, 9). No significant COVID-19-related predictors emerged for past-month alcohol or tobacco use, potentially reflecting attenuated effects of COVID-19 impacts on more proximal substance use.

Interestingly, higher COVID-19 exposure was associated with lower odds of past-year vaping, while physical impact remained positively associated with vaping. Although initially counterintuitive, this negative association with exposure may reflect fewer opportunities for social substance use behaviors due to increased isolation, stricter parental supervision, or heightened health-related caution resulting from more direct COVID-19 experiences (e.g., illness, hospitalization, or loss within families). At the same time, neither overall COVID-19 exposure nor subjective distress significantly predicted other substance use outcomes, contrasting with our original hypotheses and prior work that suggested direct links between pandemic-related stressors and adolescent substance use (2). These discrepancies might stem from methodological differences, such as the timing of assessments (initial vs. long-term pandemic responses), or unique contextual factors relevant to our adolescent chronic pain cohort. Additionally, it's possible that exposure or distress indirectly influences substance use through unmeasured variables (e.g., mental health pathways, family dynamics, and peer relationships).

Taken together, our findings—specifically the unique associations between social impact and subsequent substance use—suggest potential clinical implications for the treatment of adolescents with chronic pain. Though the ORs in the current study were modest, they still hold practical and clinical relevance for several reasons. First, given the vulnerability of this population to engaging in ineffective instrumental substance use (8, 9), identifying other potential drivers of substance use is critically important. Second, because earlier onset of substance use can be associated with more deleterious substance use trajectories, insight into developmentally specific substance use predictors is particularly relevant. Third, beyond a pandemic context, our results underscore the social salience of adolescence and suggest the clinical relevance of tailored interventions for this population. As such, more socially focused interventions for adolescents with chronic pain may help promote more effective social skills and reduce interpersonal strain and conflict. Such interventions that leverage group, peer support may be particularly effective (34). Other intervention avenues for bolstering social skills include tailored cognitive–behavioral approaches that emphasize social-perspective taking and theory of mind, which may be relevant to the adolescent pain experience and comorbid internalizing symptoms (35–37).

Limitations of the current study provide avenues for future research. The current study was limited by retrospective self-reports spanning a variable range of time after the pandemic, precluding causal inference. This study also did not examine key constructs related to adolescent substance use, such as peer substance use (38). Future studies may benefit from more comprehensive semi-structured interviewing to more thoroughly elucidate just how social impacts relate to substance use in youth with pain and from leveraging ecological–momentary assessment to better understand how these associations unfold in real time. In addition, the results of the current study cannot disentangle whether the social impact on friendships led to interpersonal strife that then prompted substance use, or whether the social impact decreased opportunities for social engagement, leading to more individual substance use. Future mechanistic studies would be best suited to examine these questions. Moreover, it should be noted that the sample analyzed in the current study was drawn from a single site, with specific social, environmental, and policy considerations, and in some analyses, the sample was quite small (e.g., logistic regressions predicting tobacco smoke), prompting the need for cautious interpretation of certain results. Finally, the sample was mostly White/non-Hispanic, thus lacking significant diversity. As such, findings should be generalized cautiously, and future studies would benefit from examining these associations in more racially, ethnically, gender, and socioeconomically diverse populations.

In conclusion, findings from the current study underscore that the social impact of COVID-19 uniquely predicted subsequent substance use, over and above mental and physical health symptoms in adolescents with chronic pain. These findings can inform socially focused interventions in multidisciplinary pain treatment and prevention efforts to best support the health and wellness of youth with chronic pain.

## Data Availability

The dataset will be made available to the principal investigator upon reasonable request.
