# Supplementary material for: Associations between COVID-19 impact and subsequent substance use in adolescents with chronic pain
Source: Front Pain Res (Lausanne). 2025 Nov 28;6:1695346. doi: 10.3389/fpain.2025.1695346 (PMC12698623; doi:10.3389/fpain.2025.1695346)
Supplement: Supplementary file 1 [file Datasheet1.docx]

***Supplementary Material***

**Associations between COVID-19 Impact and Substance Use in Adolescents with Chronic Pain**

Bridget A. Nestor PhD*, Camila Koike MD*, Kimberly Pokstis MSc, Nicole Tacugue MSc, Jack Dandaraw, Kristina Wright, Christine Greco MD, Elissa R. Weitzman ScD MSc, Lydia A. Shrier MD MPH, Joe Kossowsky PhD MMSc

**Table of Contents**

[Supplementary Figure 1. CEFIS Impact Distribution of Responses from the Physical 3](#_heading=h.q02imoqh6fnh)

[Supplementary Table 1. Demographics Characteristics 3](#_heading=h.765e067f0m78)

[Supplementary Table 2. COVID-19 Exposure Scale, % of Yes Responses 4](#_heading=h.81gwzh2v59n0)

[Supplementary Table 3. Logistic Regression Predicting Past Year Any Substance Use 6](#_heading=h.pxlfvotpamkg)

[Supplementary Table 4. Logistic Regression Predicting Past Month Any Substance Use 8](#_heading=h.57a4cjwcqx8)

[Supplementary Table 5. Logistic Regression Predicting Past Year Cannabis Use 10](#_heading=h.wbm0ka9zqkij)

[Supplementary Table 6. Logistic Regression Predicting Past Month Cannabis Use 12](#_heading=h.7h6neypb2ql)

[Supplementary Table 7. Logistic Regression Predicting Past Year Alcohol Use 14](#_heading=h.u5c30qokl87l)

[Supplementary Table 8. Logistic Regression Predicting Past Month Alcohol Use 16](#_heading=h.jhgv37ujyjo)

[Supplementary Table 9. Logistic Regression Predicting Past Year Vape Use 17](#_heading=h.m2ueno3rx2vb)

[Supplementary Table 10. Logistic Regression Predicting Past Month Vape Use 19](#_heading=h.n0lunqbqt3t2)

[Supplementary Table 11. Logistic Regression Predicting Past Year Cigarette Smoke 21](#_heading=h.hxegux5uh12m)

[Supplementary Table 12. Logistic Regression Predicting Past Month Cigarette Smoke 23](#_heading=h.rn373j2ilz8f)

[1 Supplementary Text 1. Correlation Results 23](#_heading=h.grhaqp835vs1)

# Supplementary Figure 1. CEFIS Impact Distribution of Responses from the Physical


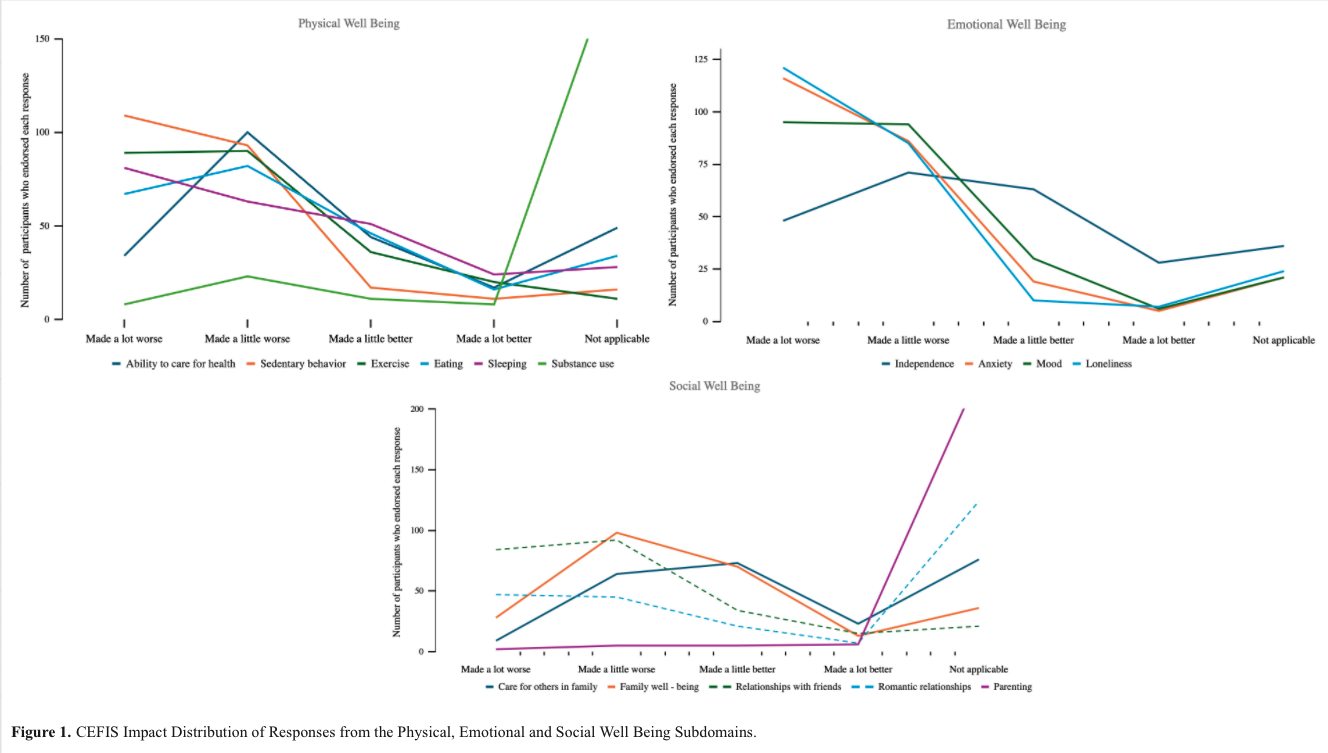


| Supplementary Table 1. Demographics Characteristics | | | | |
| --- | --- | --- | --- | --- |
|  | **Total  (N=243)** | **Non-SU  (n=139)** | **Past-Year SU (n=97)** | **Past-Month SU (n=69)** |
| Grade in school | | | | |
| 8th | 5 (2.1%) | 5 (3.6%) | 0 | 0 |
| 9th | 30 (12.3%) | 27 (19.4%) | 3 (3.1%) | 2 (2.9%) |
| 10th | 45 (18.5%) | 25 (18%) | 19 (19.6%) | 10 (14.5%) |
| 11th | 63 (25.9%) | 39 (28.1%) | 22 (22.7%) | 15 (21.7%) |
| 12th | 51 (21%) | 24 (17.3%) | 26 (26.8%) | 20 (29%) |
| 1st year undergraduate | 29 (11.9%) | 10 (7.2%) | 17 (17.5%) | 14 (20.3%) |
| 2nd year undergraduate | 10 (4.1%) | 6 (4.3%) | 4 (4.1%) | 3 (4.3%) |
| 4th year undergraduate | 1 (0.4%) | 1 (0.7%) | 0 | 0 |
| Not currently enrolled in school | 8 (3.3%) | 2 (1.4%) | 5 (5.2%) | 4 (5.8%) |
| Other | 1 (0.4%) | 0 | 1 (1%) | 1 (1.4%) |
| Parents living at home | | | | |
| One parent | 44 (18.1%) | 25 (18%) | 17 (17.5%) | 13 (18.8%) |
| Two or more parents | 198 (81.5%) | 114 (82%) | 80 (82.5%) | 56 (81.2%) |
| None | 1 (0.4%) | 0 | 0 | 0 |
| *Note: CEFIS Scores: COVID-19 Exposure and Family Impact Scales Adolescent and Young Adult Version; SU: Substance Use.* | | | | |

| Supplementary Table 2. COVID-19 Exposure Scale, % of Yes Responses | | | | | | |
| --- | --- | --- | --- | --- | --- | --- |
| **CEFIS Exposures** | **Total  (N= 243)** | **Non-SU^a, b^ (n=139)** | **Past-Year SU^a^  (n=97)** | **Past-Month SU^b^  (n=69)** | **p-value^a^** | **p-value^b^** |
| **Education** | | | | | | |
| Had a “stay at home” order | 199 (81.9%) | 115 (82.7%) | 79 (81.4%) | 56 (81.2%) | 0.94 | 0.93 |
| School was physically closed | 204 (84.6%) | 115 (83.3%) | 83 (86.5%) | 60 (87%) | 0.69 | 0.56 |
| Education was disrupted (e.g put on hold, moved to virtual learning) | 222 (91.4%) | 128 (92.1%) | 88 (90.7%) | 65 (94.2%) | 0.89 | 0.79 |
| **Family**/**Home** | | | | | | |
| Unable to visit or care for a family member | 162 (66.9%) | 98 (71%) | 58 (59.8%) | 41 (59.4%) | 0.12 | 0.15 |
| Had to start caring for a family member | 33 (13.7%) | 20 (14.4%) | 11 (11.6%) | 8 (11.8%) | 0.63 | 0.73 |
| People in family lived separately for health, safety, or job demands | 34 (14%) | 18 (12.9%) | 14 (14.4%) | 11 (15.9%) | 0.89 | 0.71 |
| Someone moved into home | 23 (9.5%) | 10 (7.2%) | 12 (12.4%) | 8 (11.6%) | 0.26 | 0.42 |
| Had to move | 13 (5.3%) | 6 (4.3%) | 7 (7.2%) | 5 (7.2%) | 0.50 | 0.58 |
| **Impact on household** | | | | | | |
| Someone in the family kept working outside the home (essential personnel) | 97 (40%) | 56 (40.3%) | 37 (38.1%) | 26 (37.7%) | 0.84 | 0.83 |
| Someone in the family/ household is a healthcare provider/ first responder providing direct care | 49 (20.2%) | 31 (22.3%) | 17 (17.7%) | 10 (14.7%) | 0.46 | 0.25 |
| Had difficulty getting food | 17 (7%) | 9 (6.5%) | 8 (8.2%) | 5 (7.2%) | 0.79 | 1.00 |
| Had difficulty getting medicine | 26 (10.7%) | 10 (7.2%) | 14 (14.4%) | 8 (11.6%) | 0.11 | 0.42 |
| Had difficulty getting healthcare when needed | 61 (25.2%) | 28 (20.1%) | 28 (29.2%) | 20 (29.4%) | 0.16 | 0.21 |
| Had difficulty getting other essentials (e.g., cleaning supplies, masks, etc.) | 37 (15.4%) | 22 (16.1%) | 14 (14.4%) | 8 (11.6%) | 0.91 | 0.54 |
| Self - quarantined due to travel or possible exposure | 180 (74.1%) | 100 (71.9%) | 76 (78.4%) | 53 (76.8%) | 0.34 | 0.56 |
| Income decreased | 71 (29.3%) | 34 (24.6%) | 34 (35.1%) | 25 (36.2%) | 0.11 | 0.11 |
| Had to cut back hours at work | 29 (12%) | 9 (6.5%) | 18 (18.6%) | 13 (18.8%) | **0.01*** | **0.01*** |
| Member of the family had to cut back hours at work | 62 (25.6%) | 34 (24.6%) | 26 (26.8%) | 19 (27.5%) | 0.80 | 0.76 |
| Required to stop working (expect to be called back) | 7 (2.9%) | 3 (2.2%) | 4 (4.1%) | 3 (4.3%) | 0.63 | 0.65 |
| **Disruptions and losses** | | | | | | |
| Member of the family was required to stop working (expect to be called back) | 35 (14.5%) | 19 (13.8%) | 14 (14.4%) | 12 (17.4%) | 1.00 | 0.62 |
| Lost job permanently | 2 (0.8%) | 0 | 2 (2.1%) | 2 (2.9%) | 0.33 | 0.21 |
| Member of the family lost job permanently | 10 (4.1%) | 6 (4.3%) | 4 (4.1%) | 3 (4.3%) | 1.00 | 1.00 |
| Lost health insurance/ benefits | 1 (0.4%) | 0 | 1 (1%) | 1 (1.4%) | 0.86 | 0.72 |
| Member of family lost their health insurance/ benefits | 4 (1.7%) | 3 (2.2%) | 1 (1%) | 1 (1.4%) | 0.88 | 1.00 |
| Missed an important milestone event that was cancelled or postponed (e.g., graduation, prom, wedding) | 126 (52.1%) | 72 (52.2%) | 52 (53.6%) | 35 (50.7%) | 0.89 | 1.00 |
| Missed an important family event or it was cancelled (e.g., birth, funeral, travel [including vacation]) | 154 (64.2%) | 88 (64.2%) | 62 (64.6%) | 45 (65.2%) | 1.00 | 0.91 |
| **Health impact and loss** | | | | | | |
| Someone in the family was exposed to someone with COVID-19 | 205 (84.4%) | 114 (82%) | 86 (88.7%) | 61 (88.4%) | 0.23 | 0.32 |
| Someone in the family had symptoms or was diagnosed with COVID-19 | 202 (83.1%) | 119 (85.6%) | 78 (80.4%) | 54 (78.3%) | 0.38 | 0.26 |
| Someone in the family tried to get tested for COVID-19, but couldn’t | 26 (10.7%) | 14 (10.1%) | 12 (12.4%) | 9 (13%) | 0.73 | 0.68 |
| Someone in the family was hospitalized from COVID-19 | 27 (11.1%) | 12 (8.6%) | 14 (14.4%) | 9 (13%) | 0.23 | 0.45 |
| Someone in the family was in the intensive Care Unit for COVID-19 | 10 (4.1%) | 4 (2.9%) | 6 (6.2%) | 4 (5.8%) | 0.36 | 0.52 |
| Someone in the family died from COVID-19 | 19 (7.8%) | 10 (7.2%) | 9 (9.3%) | 8 (11.6%) | 0.74 | 0.42 |
| *Note: SU: Substance Use; N: total population; n:population.  Significant p-value<.05.*  *CEFIS Scores: COVID-19 Exposure and Family Impact Scales Adolescent and Young Adult Version;  a: Past-Year SU vs Non-SU*  *a: Past-Month SU vs Non-SU* | | | | | | |

| Supplementary Table 3. Logistic Regression Predicting Past Year Any Substance Use | | | | | |
| --- | --- | --- | --- | --- | --- |
|  | **OR/Wald** | **95% CI** | **SE** | **p-value** | **Nagelkerke R Square** |
| **Model 0** | | | | | .119 |
| CEFIS Exposure Total Score | 1.005 | [.926, 1.090] | .041 | .910 |  |
| CEFIS Impact Total Score | 1.077 | [1.038, 1.117] | .019 | <.001 |  |
| CEFIS Distress Total Score | .947 | [.819, 1.095] | .074 | .460 |  |
| **Model 1** | | | | | .132 |
| CEFIS Impact Physical | 1.046 | [.962, 1.136] | .042 | .294 |  |
| CEFIS Impact Emotional | .991 | [.882, 1.113] | .059 | .877 |  |
| CEFIS Impact Social | 1.200 | [1.083, 1.330] | .052 | <.001 |  |
| **Model 2** | | | | | .233 |
| CEFIS Impact Social | 1.218 | [1.109, 1.337 | .048 | <.001 |  |
| Age | 1.476 | [1.188, 1.835] | .111 | <.001 |  |
| Gender | 4.383 |  |  | .357 |  |
| **Model 3** | | | | | .226 |
| CEFIS Impact Social | 1.194 | [1.082, 1.318] | .050 | <.001 |  |
| Age | 1.538 | [1.228, 1.925] | .115 | <.001 |  |
| Anxiety | .943 | [.600, 1.484] | .231 | .801 |  |
| Depression | 1.145 | [.705, 1.858] | .247 | .584 |  |
| Stress | 1.020 | [.970, 1.072] | .026 | .449 |  |
| **Model 4** | | | | | .244 |
| CEFIS Impact Social | 1.248 | [1.131, 1.377] | .050 | <.001 |  |
| Age | 1.452 | [1.156, 1.824] | .116 | .001 |  |
| Pain | .863 | [.701, 1.062] | .106 | .164 |  |
| Pain Interference | 1.283 | [.765, 2.151] | .264 | .344 |  |
| Functional Disability | .982 | [.943, 1.022] | .021 | .371 |  |
| *Note: OR reported for continuous variables. Wald reported for Gender as a categorical variable. CEFIS Scores: COVID-19 Exposure and Family Impact Scales Adolescent and Young Adult Version.* | | | | | |

| \| Supplementary Table 4. Logistic Regression Predicting Past Month Any Substance Use \| \| \| \| \| \| \| --- \| --- \| --- \| --- \| --- \| --- \| \|  \| **OR** \| **95% CI** \| **SE** \| **p-value** \| **Nagelkerke R Square** \| \| **Model 0** \| \| \| \| \| .134 \| \| CEFIS Exposure Total Score \| .995 \| [.911, 1.087] \| .045 \| .911 \|  \| \| CEFIS Impact Total Score \| 1.095 \| [1.049, 1.143] \| .022 \| <.001 \|  \| \| CEFIS Distress Total Score \| .913 \| [.777, 1.072] \| .082 \| .264 \|  \| \| **Model 1** \| \| \| \| \| .136 \| \| CEFIS Impact Physical \| 1.068 \| [.974, 1.171] \| .047 \| .163 \|  \| \| CEFIS Impact Emotional \| .969 \| [.852, 1.102] \| .066 \| .628 \|  \| \| CEFIS Impact Social \| 1.207 \| [1.080, 1.348] \| .057 \| <.001 \|  \| \| **Model 2** \| \| \| \| \| .239 \| \| CEFIS Impact Social \| 1.240 \| [1.120, 1.374] \| .052 \| <.001 \|  \| \| Age \| 1.623 \| [1.267, 2.080] \| .126 \| <.001 \|  \| \| Gender \| 2.949 \|  \|  \| .566 \|  \| \| **Model 3** \| \| \| \| \| .238 \| \| CEFIS Impact Social \| 1.209 \| [1.086, 1.346] \| .055 \| <.001 \|  \| \| Age \| 1.676 \| [1.293, 2.173] \| .132 \| <.001 \|  \| \| Anxiety \| .879 \| [.532, 1.453] \| .257 \| .615 \|  \| \| Depression \| 1.249 \| [.732, 2.131] \| .273 \| .415 \|  \| \| Stress \| 1.016 \| [.961, 1.073] \| .028 \| .585 \|  \| \| **Model 4** \| \| \| \| \| .255 \| \| CEFIS Impact Social \| 1.274 \| [1.144, 1.419 \| .055 \| <.001 \|  \| \| Age \| 1.661 \| [1.279, 2.156] \| .133 \| <.001 \|  \| \| Pain \| 1.055 \| [.843, 1.319] \| .114 \| .642 \|  \| \| Pain Interference \| 1.159 \| [.662, 2.029] \| .286 \| .605 \|  \| \| Functional Disability \| .973 \| [.931, 1.017] \| .023 \| .224 \|  \| \| *Note: OR reported for continuous variables. Wald reported for Gender as a categorical variable. CEFIS Scores: COVID-19 Exposure and Family Impact Scales Adolescent and Young Adult Version.* \| \| \| \| \| \| |
| --- | --- | --- | --- | --- | --- | --- | --- | --- | --- | --- | --- | --- | --- | --- | --- | --- | --- | --- | --- | --- | --- | --- | --- | --- | --- | --- | --- | --- | --- | --- | --- | --- | --- | --- | --- | --- | --- | --- | --- | --- | --- | --- | --- | --- | --- | --- | --- | --- | --- | --- | --- | --- | --- | --- | --- | --- | --- | --- | --- | --- | --- | --- | --- | --- | --- | --- | --- | --- | --- | --- | --- | --- | --- | --- | --- | --- | --- | --- | --- | --- | --- | --- | --- | --- | --- | --- | --- | --- | --- | --- | --- | --- | --- | --- | --- | --- | --- | --- | --- | --- | --- | --- | --- | --- | --- | --- | --- | --- | --- | --- | --- | --- | --- | --- | --- | --- | --- | --- | --- | --- | --- | --- | --- | --- | --- | --- | --- | --- | --- | --- | --- | --- | --- | --- | --- | --- | --- | --- | --- | --- | --- | --- | --- | --- | --- | --- | --- | --- | --- | --- | --- | --- | --- | --- | --- | --- | --- | --- | --- | --- | --- | --- |

| Supplementary Table 5. Logistic Regression Predicting Past Year Cannabis Use | | | | | |
| --- | --- | --- | --- | --- | --- |
|  | **OR** | **95% CI** | **SE** | **p-value** | **Nagelkerke R Square** |
| **Model 0** | | | | | .181 |
| CEFIS Exposure Total Score | .983 | [.895, 1.080] | .048 | .723 |  |
| CEFIS Impact Total Score | 1.127 | [1.071, 1.185] | .026 | <.001 |  |
| CEFIS Distress Total Score | .869 | [.730, 1.035] | .089 | .116 |  |
| **Model 1** | | | | | .151 |
| CEFIS Impact Physical | 1.111 | [1.005,1.228] | .051 | .039 |  |
| CEFIS Impact Emotional | 1.035 | [.898, 1.193] | .072 | .630 |  |
| CEFIS Impact Social | 1.149 | [1.026, 1.287] | .058 | .016 |  |
| **Model 2** | | | | | .263 |
| CEFIS Impact Physical | 1.129 | [1.028, 1.240] | .048 | .011 |  |
| CEFIS Impact Social | 1.139 | [1.013, 1.281] | .060 | .030 |  |
| Age | 1.504 | [1.155, 1.957] | .135 | .002 |  |
| Gender | 9.322 |  |  | .054 |  |
| **Model 3** | | | | | .232 |
| CEFIS Impact Physical | 1.114 | [1.016,1.222] | .047 | .021 |  |
| CEFIS Impact Social | 1.139 | [1.010,1.284] | .061 | .033 |  |
| Age | 1.542 | [1.174, 2.026] | .139 | .002 |  |
| Anxiety | .644 | [.377, 1.099] | .273 | .107 |  |
| Depression | 1.414 | [.797, 2.508] | .293 | .237 |  |
| Stress | 1.020 | [.962, 1.082] | .030 | .510 |  |
| **Model 4** | | | | | .216 |
| CEFIS Impact Physical | 1.111 | [1.011, 1.220] | .048 | .028 |  |
| CEFIS Impact Social | 1.143 | [1.019, 1.282] | .058 | .022 |  |
| Age | 1.464 | [1.127, 1.903] | .134 | .004 |  |
| Pain | .927 | [.731, 1.176] | .121 | .533 |  |
| Pain Interference | 1.298 | [.721, 2.338] | .300 | .385 |  |
| Functional Disability | .987 | [.944, 1.032] | .023 | .573 |  |
| *Note: OR reported for continuous variables. Wald reported for Gender as a categorical variable. CEFIS Scores: COVID-19 Exposure and Family Impact Scales Adolescent and Young Adult Version.* | | | | | |

| Supplementary Table 6. Logistic Regression Predicting Past Month Cannabis Use | | | | | |
| --- | --- | --- | --- | --- | --- |
|  | **OR** | **95% CI** | **SE** | **p-value** | **Nagelkerke R Square** |
| **Model 0** | | | | | .194 |
| CEFIS Exposure Total Score | 1.047 | [.939, 1.167] | .056 | .413 |  |
| CEFIS Impact Total Score | 1.135 | [1.070, 1.205] | .030 | <.001 |  |
| CEFIS Distress Total Score | .902 | [.739, 1.099] | .101 | .306 |  |
| **Model 1** | | | | | .185 |
| CEFIS Impact Physical | 1.195 | [1.057, 1.351] | .063 | .004 |  |
| CEFIS Impact Emotional | .997 | [.845, 1.177] | .085 | .972 |  |
| CEFIS Impact Social | 1.147 | [1.009, 1.304] | .065 | .035 |  |
| **Model 2** | | | | | .265 |
| CEFIS Impact Physical | 1.189 | [1.061, 1.334] | .058 | .003 |  |
| CEFIS Impact Social | 1.141 | [.999, 1.303] | .068 | .051 |  |
| Age | 1.505 | [1.106, 2.047] | .157 | .009 |  |
| Gender | 5.844 |  |  | .211 |  |
| **Model 3** | | | | | .232 |
| CEFIS Impact Physical | 1.218 | [1.093, 1.357] | .055 | <.001 |  |
| Age | 1.512 | [1.093, 2.052] | .156 | .008 |  |
| Anxiety | 1.131 | [.619, 2.069] | .308 | .689 |  |
| Depression | 1.588 | [.824, 3.057] | .334 | .167 |  |
| Stress | .981 | [.918, 1.048] | .034 | .565 |  |
| **Model 4** | | | | | .221 |
| CEFIS Impact Physical | 1.226 | [1.100, 1.366] | .055 | <.001 |  |
| Age | 1.454 | [1.078, 1.960] | .153 | .014 |  |
| Pain | .962 | [.734, 1.259] | .138 | .775 |  |
| Pain Interference | 1.604 | [.821, 3.133] | .342 | .167 |  |
| Functional Disability | .976 | [.928, 1.026] | .026 | .339 |  |
| *Note: OR reported for continuous variables. Wald reported for Gender as a categorical variable. CEFIS Scores: COVID-19 Exposure and Family Impact Scales Adolescent and Young Adult Version.* | | | | | |

| Supplementary Table 7. Logistic Regression Predicting Past Year Alcohol Use | | | | | |
| --- | --- | --- | --- | --- | --- |
|  | **OR** | **95% CI** | **SE** | **p-value** | **Nagelkerke R Square** |
| **Model 0** | | | | | .086 |
| CEFIS Exposure Total Score | .949 | [.872, 1.033] | .043 | .226 |  |
| CEFIS Impact Total Score | 1.069 | [1.029, 1.110] | .019 | <.001 |  |
| CEFIS Distress Total Score | .956 | [.821, 1.113] | .078 | .563 |  |
| **Model 1** | | | | | .087 |
| CEFIS Impact Physical | 1.040 | [.953, 1.134] | .044 | .376 |  |
| CEFIS Impact Emotional | .948 | [.841, 1.069] | .061 | .384 |  |
| CEFIS Impact Social | 1.176 | [1.058, 1.307] | .054 | .003 |  |
| **Model 2** | | | | | .166 |
| CEFIS Impact Social | 1.181 | [1.076, 1.296] | .047 | <.001 |  |
| Age | 1.470 | [1.174, 1.841] | .115 | <.001 |  |
| Gender | .752 |  |  | .945 |  |
| **Model 3** | | | | | .181 |
| CEFIS Impact Social | 1.148 | [1.039, 1.268] | .051 | .007 |  |
| Age | 1.544 | [1.217, 1.959] | .121 | <.001 |  |
| Anxiety | .812 | [.505, 1.304] | .242 | .389 |  |
| Depression | 1.216 | [.735, 2.011] | .257 | .446 |  |
| Stress | 1.027 | [.974, 1.082] | .027 | .322 |  |
| **Model 4** | | | | | .216 |
| CEFIS Impact Social | 1.223 | [1.106, 1.353] | .051 | <.001 |  |
| Age | 1.510 | [1.186, 1.922] | .123 | <.001 |  |
| Pain | 1.047 | [.847, 1.294] | .108 | .671 |  |
| Pain Interference | 1.262 | [.739, 2.157] | .273 | .394 |  |
| Functional Disability | .949 | [.908, .991] | .022 | .017 |  |
| *Note: OR reported for continuous variables. Wald reported for Gender as a categorical variable. CEFIS Scores: COVID-19 Exposure and Family Impact Scales Adolescent and Young Adult Version.* | | | | | |

| Supplementary Table 8. Logistic Regression Predicting Past Month Alcohol Use | | | | | |
| --- | --- | --- | --- | --- | --- |
|  | **OR** | **95% CI** | **SE** | **p-value** | **Nagelkerke R Square** |
| **Model 0** | | | | | .039 |
| CEFIS Exposure Total Score | 1.022 | [.919, 1.137] | .054 | .691 |  |
| CEFIS Impact Total Score | 1.047 | [1.002, 1.095] | .023 | .041 |  |
| CEFIS Distress Total Score | .858 | [.709, 1.038] | .097 | .114 |  |
| **Model 1** | | | | | .030 |
| CEFIS Impact Physical | 1.008 | [.907, 1.119] | .054 | .885 |  |
| CEFIS Impact Emotional | .950 | [.823, 1.097] | .073 | .486 |  |
| CEFIS Impact Social | 1.126 | [.993, 1.276] | .064 | .065 |  |
| *Note: OR reported for continuous variables. CEFIS Scores: COVID-19 Exposure and Family Impact Scales Adolescent and Young Adult Version.* | | | | | |

| Supplementary Table 9. Logistic Regression Predicting Past Year Vape Use | | | | | |
| --- | --- | --- | --- | --- | --- |
|  | **OR** | **95% CI** | **SE** | **p-value** | **Nagelkerke R Square** |
| **Model 0** | | | | | .151 |
| CEFIS Exposure Total Score | .891 | [.797, .995] | .057 | .041 |  |
| CEFIS Impact Total Score | 1.118 | [1.051, 1.189] | .032 | <.001 |  |
| CEFIS Distress Total Score | 1.005 | [.815, 1.239] | .107 | .965 |  |
| **Model 1** | | | | | .200 |
| CEFIS Exposure Total | .871 | [.773, .981] | .061 | .023 |  |
| CEFIS Impact Physical | 1.228 | [1.064, 1.418] | .073 | .005 |  |
| CEFIS Impact Emotional | 1.004 | [.835, 1.207] | .094 | .966 |  |
| CEFIS Impact Social | 1.161 | [1.007, 1.339] | .073 | .040 |  |
| **Model 2** | | | | | .265 |
| CEFIS Exposure Total | .870 | [.770, .982] | .062 | .024 |  |
| CEFIS Impact Physical | 1.220 | [1.065, 1.397] | .069 | .004 |  |
| Age | 1.441 | [1.025, 2.025] | .174 | .036 |  |
| Gender | 3.145 |  |  | .534 |  |
| **Model 3** | | | | | .226 |
| CEFIS Exposure Total | .865 | [.765, .977] | .062 | .020 |  |
| CEFIS Impact Physical | 1.218 | [1.082, 1.372] | .061 | .001 |  |
| Age | 1.452 | [1.037, 2.034] | .172 | .030 |  |
| Anxiety | .606 | [.320, 1.150] | .327 | .126 |  |
| Depression | 2.356 | [1.097, 5.061] | .390 | .028 |  |
| Stress | 1.005 | [.934, 1.080] | .037 | .902 |  |
| **Model 4** | | | | | .213 |
| CEFIS Exposure Total | .874 | [.775, .985] | .061 | .028 |  |
| CEFIS Impact Physical | 1.217 | [1.075, 1.377] | .063 | .002 |  |
| Age | 1.455 | [1.045, 2.027] | .169 | .027 |  |
| Pain | 1.140 | [.845, 1.537] | .153 | .393 |  |
| Pain Interference | 1.041 | [.489, 2.215] | .386 | .918 |  |
| Functional Disability | .976 | [.918, 1.037] | .031 | .431 |  |
| Depression | 1.768 | [.996, 3.140] | .293 | .052 |  |
| *Note: OR reported for continuous variables. Wald reported for Gender as a categorical variable. CEFIS Scores: COVID-19 Exposure and Family Impact Scales Adolescent and Young Adult Version.* | | | | | |

| Supplementary Table 10. Logistic Regression Predicting Past Month Vape Use | | | | | |
| --- | --- | --- | --- | --- | --- |
|  | **OR** | **95% CI** | **SE** | **p-value** | **Nagelkerke R Square** |
| **Model 0** | | | | | .326 |
| CEFIS Exposure Total Score | .866 | [.751, 1.000] | .073 | .050 |  |
| CEFIS Impact Total Score | 1.237 | [1.105, 1.386] | .058 | <.001 |  |
| CEFIS Distress Total Score | 1.293 | [.953, 1.754] | .156 | .099 |  |
| **Model 1** |  |  |  |  | .276 |
| CEFIS Impact Physical | 1.352 | [1.090, 1.677] | .110 | .006 |  |
| CEFIS Impact Emotional | 1.047 | [.796, 1.377] | .140 | .743 |  |
| CEFIS Impact Social | 1.223 | [1.006, 1.487] | .100 | .044 |  |
| **Model 2** |  |  |  |  | .297 |
| CEFIS Impact Physical | 1.372 | [1.115, 1.690] | .106 | .003 |  |
| CEFIS Impact Social | 1.218 | [1.002, 1.482] | .100 | .048 |  |
| Age | 1.253 | [.787, 1.996] | .238 | .342 |  |
| Gender | .431 |  |  | .980 |  |
| **Model 3** |  |  |  |  | .328 |
| CEFIS Impact Physical | 1.353 | [1.098, 1.666] | .106 | .005 |  |
| CEFIS Impact Social | 1.197 | [.982, 1.459] | .101 | .075 |  |
| Anxiety | .578 | [.225, 1.486] | .482 | .255 |  |
| Depression | 2.877 | [.901, 9.188] | .592 | .074 |  |
| Stress | 1.018 | [.924, 1.122] | .049 | .716 |  |
| **Model 4** |  |  |  |  | .271 |
| CEFIS Impact Physical | 1.498 | [1.220, 1.840] | .105 | <.001 |  |
| Pain | 1.452 | [.951, 2.219] | .216 | .084 |  |
| Pain Interference | 1.120 | [.442, 2.839] | .475 | .811 |  |
| Functional Disability | .990 | [.921, 1.064] | .037 | .782 |  |
| *Note: OR reported for continuous variables. Wald reported for Gender as a categorical variable. CEFIS Scores: COVID-19 Exposure and Family Impact Scales Adolescent and Young Adult Version.* | | | | | |

| Supplementary Table 11. Logistic Regression Predicting Past Year Cigarette Smoke | | | | | |
| --- | --- | --- | --- | --- | --- |
|  | **OR** | **95% CI** | **SE** | **p-value** | **Nagelkerke R Square** |
| **Model 0** | | | | | .148 |
| CEFIS Exposure Total Score | .999 | [.844, 1.182] | .086 | .989 |  |
| CEFIS Impact Total Score | 1.133 | [1.020, 1.260] | .054 | .020 |  |
| CEFIS Distress Total Score | 1.114 | [.791, 1.568] | .175 | .537 |  |
| **Model 1** | | | | | .157 |
| CEFIS Impact Physical | 1.155 | [.927, 1.438] | .112 | .200 |  |
| CEFIS Impact Emotional | .967 | [.720, 1.299] | .150 | .824 |  |
| CEFIS Impact Social | 1.263 | [1.005, 1.588] | .117 | .045 |  |
| **Model 2** | | | | | .215 |
| CEFIS Impact Social | 1.335 | [1.087, 1.639] | .105 | .006 |  |
| Age | 1.404 | [.825, 2.390] | .271 | .212 |  |
| Gender | 2.593 |  |  | .628 |  |
| **Model 3** | | | | | .196 |
| CEFIS Impact Social | 1.352 | [1.092, 1.674] | .109 | .006 |  |
| Anxiety | .621 | [.214, 1.799] | .543 | .380 |  |
| Depression | 3.687 | [.979, 13.881] | .676 | .054 |  |
| Stress | .929 | [.827, 1.043] | .059 | .212 |  |
| **Model 4** | | | | | .160 |
| CEFIS Impact Social | 1.339 | [1.089, 1.647] | .106 | .006 |  |
| Pain | .894 | [.566,1.414] | .234 | .633 |  |
| Pain Interference | 2.046 | [.691, 6.054] | .554 | .196 |  |
| Functional Disability | .984 | [.902, 1.072] | .044 | .709 |  |
| *Note: OR reported for continuous variables. Wald reported for Gender as a categorical variable. CEFIS Scores: COVID-19 Exposure and Family Impact Scales Adolescent and Young Adult Version.* | | | | | |

| Supplementary Table 12. Logistic Regression Predicting Past Month Cigarette Smoke | | | | | |
| --- | --- | --- | --- | --- | --- |
|  | **OR** | **95% CI** | **SE** | **p-value** | **Nagelkerke R Square** |
| **Model 0** | | | | | .298 |
| CEFIS Exposure Total Score | .834 | [.643, 1.081] | .132 | .171 |  |
| CEFIS Impact Total Score | 1.266 | [.987, 1.624] | .127 | .064 |  |
| CEFIS Distress Total Score | 1.433 | [.695, 2.953] | .369 | .329 |  |
| *Note: OR reported for continuous variables. CEFIS Scores: COVID-19 Exposure and Family Impact Scales Adolescent and Young Adult Version.* | | | | | |

# Supplementary Text 1. Correlation Results

Pain interference scores were positively associated with CEFIS exposure (r=0.18, p=0.01), CEFIS distress (r=0.30, p<0.01), CEFIS impact emotional (r=0.32, p<0.01), CEFIS impact physical (r=0.26, p<0.01), CEFIS impact social (r=0.17, p=0.03), and overall CEFIS impact (r=0.31, p<0.01). Functional disability was positively associated with CEFIS exposure (r=0.25, p<0.01), CEFIS distress (r=0.25, p<0.01), CEFIS impact emotional (r=0.31, p<0.01), CEFIS impact physical (r=0.24, p<0.01), CEFIS impact social (r=0.16, p=0.04), and overall CEFIS impact (r=0.29, p<0.01).

Anxiety symptoms were positively associated with CEFIS exposure (r=0.23, p<0.01), CEFIS distress (r=0.36, p<0.01), CEFIS impact emotional (r=0.40, p<0.01), CEFIS impact physical (r=0.28, p<0.01), CEFIS impact social (r=0.37, p<0.01), and overall CEFIS impact (r=0.42, p<0.01). Depressive symptoms were positively associated with CEFIS exposure (r=0.20, p<0.01), CEFIS distress (r=0.28, p<0.01), CEFIS impact emotional (r=0.40, p<0.01), CEFIS impact physical (r=0.30, p<0.01), CEFIS impact social (r=0.38, p<0.01), and overall CEFIS impact (r=0.44, p<0.01). Psychological stress was positively associated with CEFIS exposure (r=0.16, p=0.03), CEFIS distress (r=0.26, p<0.01), CEFIS impact emotional (r=0.41, p<0.01), CEFIS impact physical (r=0.32, p<0.01), CEFIS impact social (r=0.36, p<0.01), and overall CEFIS impact (r=0.44, p<0.01).

Other significant positive associations were identified among measures of substance use, pain, and mental health.
